# Excessive Dpp signaling induces cardial apoptosis through dTAK1 and dJNK during late embryogenesis of *Drosophila*

**DOI:** 10.1186/1423-0127-18-85

**Published:** 2011-11-24

**Authors:** Sheng-An Yang, Ming-Tsan Su

**Affiliations:** 1 Department of Life Science, National Taiwan Normal University, Taipei 11677, Taiwan

## Abstract

**Background:**

To identify genes involved in the heart development of *Drosophila*, we found that embryos lacking *raw *function exhibited cardial phenotypes. *raw *was initially identified as a dorsal open group gene. The dorsal open phenotype was demonstrated to be resulted from the aberrant expression of *decapentaplegic *(*dpp*), a member of the tumor growth factor beta (TGF-β), signaling pathway. Despite the role of *dpp *in pattering cardioblasts during early embryogenesis of *Drosophila *have been demonstrated, how mutation in *raw *and/or excessive *dpp *signaling involves in the differentiating heart of *Drosophila *has not been fully elaborated at late stages.

**Results:**

We show that *raw *mutation produced a mild overspecification of cardial cells at stage 14, but these overproduced cells were mostly eliminated in late mutant embryos due to apoptosis. Aberrant *dpp *signaling is likely to contribute to the cardial phenotype found in *raw *mutants, because expression of *dpp *or constitutively activated *thickven *(*tkv^CA^*), the type I receptor of Dpp, induced a *raw*-like phenotype. Additionally, we show that *dpp *induced non-autonomous apoptosis through TGFβ activated kinase 1 (*TAK1*), because mis-expression of a dominant negative form of *Drosophila TAK1 *(*dTAK1^DN^*) was able to suppress cell death in *raw *mutants or embryos overexpressing *dpp*. Importantly, we demonstrated that *dpp *induce its own expression through *dTAK1*, which also leads to the hyperactivation of *Drosophila *JNK (DJNK). The hyperactivated DJNK was attributed to be the cause of Dpp/DTAK1-induced apoptosis because overexpression of a dominant negative DJNK, *basket *(*bsk^DN^*), suppressed cell death induced by Dpp or DTAK1. Moreover, targeted overexpression of the anti-apoptotic P35 protein, or a dominant negative proapoptotic P53 (P53^DN^) protein blocked Dpp/DTAK1-induced apoptosis, and rescued heart cells under the *raw *mutation background.

**Conclusions:**

We find that ectopic Dpp led to DJNK-dependent cardial apoptosis through the non-canonical TGF-β pathway during late embryogenesis of *Drosophila*. This certainly will increase our understanding of the pathogenesis of cardiomyopathy, because haemodynamic overload can up-regulate TGF-β and death of cardiomyocytes is observed in virtually every myocardial disease. Thus, our study may provide possible medical intervention for human cardiomyopathy.

## Background

The *Drosophila *heart is a simple tubular organ located at the dorsal midline beneath the epidermis, and it is therefore alternatively termed the dorsal vessel. The fly heart consists of two major cell types, myocardial cells and pericardial cells, which arise from two bilateral rows of cardiac primordia at the leading edge of the migrating mesoderm. The contractile myocardial cells which form the lumen are arranged in a segmental repeat comprised of six cells per hemisegment in the mature embryonic heart. The pericardial cells, which are essential for normal cardiac function, are aligned alongside the myocardial cells. Despite its simple structure, fly heart has recently emerged as an excellent model system for dissecting the complex pathway that determines cardiogenic cell fate, and for investigating the physiologic function of the adult heart [1,2].

Extensive study has revealed that a combinatory action of extrinsic signaling and intrinsic transcription network is required for correct specification of cardial precursors and differentiation of mature heart (reviewed in [3]). Of all external signalings, Dpp, a member of the mammalian Transforming growth factor superfamily (TGF-β), has been shown to play a pivotal role during cardiogenesis of *Drosophila *[4]. The cardiogenic function of Dpp begins when it is expressed in the dorsal epidermis in a broad band along the anterior-posterior axis during germ band extension in *Drosophila *[5]. This spatiotemporal pattern of Dpp specifies the underling dorsal mesodermal cell fate by maintaining the expression of the transcription factor, *tinman *(*tin*) [4,6-8]. Dpp also regulates the expression of several other cardiogenic transcription factors, including *pannier *(*pnr*) and *dorsocross *(*doc*) [9,10]. For further specification of the cardiogenic mesoderm, Wg signaling together with the combinatorial action of several transcription factors, including *tin*, *pnr*, *doc *and *tailup*, are required [11-20]. Around stage 10, Dpp expression in the dorsal ectoderm vanishes briefly, but reappears in the leading edge (LE) cells of the dorsal ectoderm at stage 11. This second round of Dpp expression in LE cells persists through stage 17 [21]. Interestingly, pMad, the activated Dpp signal transducer, can be detected in a subset of cardial progenitors in stages 12 to 14 [22]. This indicates that a second round of Dpp activity is required for further differentiation of *Drosophila *heart. Indeed, *dpp *mutants with alleles that affect the expression of Dpp in LE cells have impaired embryonic heart development and larval cardiac function [23,24]. These findings indicate a biphasic requirement for Dpp during cardiogenesis of *Drosophila*, in which it is required early for dorsal mesoderm patterning and later for differentiating heart cells.

Dpp regulates many developmental processes, including cell fate determination, alteration of cell shape, proliferation, and apoptosis. Morphogenic function of Dpp in cell fate determination has been shown to be mediated through the canonical pathway, in which it interacts with a type I receptor, Tkv, and a type II receptor, Punt. Upon formation of ligand-receptor complex, activated Punt phosphorylates Mad, which subsequently interacts with Medea. The resultant complex containing pMad, and Medea is then translocated into the nucleus where it activates transcription of Dpp target genes [25]. Other than the canonical pathway, it has been found that mammalian Dpp homolog, TGF-β transduce its signaling that is independent of Smad, a homolog of *Drosophila *pMad. The Smad-independent pathway is designated as the non-canonical TGF-β pathway. In the non-canonical pathway, TGF-β activated kinase 1 (TAK1) forms a multiple protein complex protein complex with TRAF6, TAB2, and TAB3. Upon binding of TGF-β to its receptor, TRAF6 exerts its E3 ubiquitin ligase activity together with ubiquitin-conjugating enzymes to catalyze Lys63-linked polyubiquitination. Subsequently, the Lys63-linked polyubiquitin chain associates with TAB2 which leads to autophosphorylation and activation of TAK1 [26,27]. Despite it is less clear in *Drosophila*, many functionally-conserved non-canonical signaling transducers, including DTAK1 and TAB2, have been identified in *Drosophila *[28,29]. Moreover, Dpp signaling has also been shown to control the viability of cells. A lack of Dpp signaling activates c-Jun N-terminal kinase (JNK)-dependent apoptosis in wing discs [30]. Interestingly, Dpp is also likely to function as a pro-apoptotic signal because increased Dpp activity leads to both non-autonomous JNK activation and cell death [31]. However, the link between Dpp signaling and JNK-mediated apoptosis is currently unclear in *Drosophila*.

Dpp is a downstream target of the JNK pathway, a conserved and pleiotropic signaling system whose function governs many different biological activities, including morphogenesis, differentiation, proliferation and apoptosis. Components of the DJNK pathway, including Djun and Raw, have been shown to participate in *Drosophila *heart development by modulating the expression of Dpp [14,16]. In *Djun *mutant embryos, the expression of *dpp *is not maintained at dorsal edge, which leads to down-regulation of cardiac *tin *at later stages [16]. By contrast, pericardial cells are overspecified in *raw *loss-of-function mutant embryos [14]. The excessive differentiation of cardial cells has been attributed to the ectopic Dpp activity induced by dysregulated DJNK signaling.

In our effort to identify genes involved in the heart development of *Drosophila*, we observed that embryos lacking *raw *function exhibited cardial phenotypes, in which heart cells were overspecified in moderately degree during mid-embryogenesis, and that the overproduced heart cells had disappeared at late stages. We show here that the elimination of heart cells in late *raw *mutant embryos was a result of excessive apoptosis, and ectopic Dpp signaling was responsible for the cardial apoptosis phenotypes of *raw *mutant embryos. We also found that elevated Dpp can function as a pro-apoptotic signal to promote non-autonomous apoptosis in a dose-dependent manner. Importantly, ectopic Dpp auto-regulate its own expression through DTAK1. The autocrine Dpp further enhanced the expression of DJNK and consequently led to P53-dependent cell death. Our study defined a novel pathway which linked ectopic Dpp signaling and DJNK-dependent apoptosis during late cardiogenesis of *Drosophila*.

## Methods

### Fly stocks and genetics

Fly stocks were raised and crossed at 25°C. Gal4 drivers: *Act5C-gal4 *(constitutive), *24B-gal4 *(mesodermal), and *69B-gal4 *(ectodermal) were obtained from the Bloomington Stock Center. The *svp *specific enhancer trap line E2-3-9 has been described [32]. *him-*GFP, a reporter in which a 2.2 kb genomic fragment spanning the promoter region and 5' untranslated region of *him *was placed upstream of nuclear green fluorescent protein encoded gene reporter, was used to mark the muscle, cardial and pericardial cells [33], The *dpp-*LacZ BS3.0 reporter reflects the transcription of *dpp *in embryos [34]. *raw^1 ^*has been described [35]. UAS-*dpp*, UAS-*bsk*, UAS-*bsk^DN^*, UAS-*p35 *[36]; UAS-*p53*, UAS-*p53^DN^*, UAS-*brk*, *tkv^7 ^*and *tkv^CA ^*were also obtained from the Bloomington Stock Center. UAS-*tkv-RNAi *(102319, obtained from the Vienna *Drosophila *RNAi Center, VDRC); UAS-*dTAK1 *and UAS-*dTAK1^DN ^*[29] were gifts from T. Adachi-Yamada. The isogenic *w^1118 ^*flies were used as wild-type (WT) controls.

### Plasmid construction and generation of transgenic flies

The UAS/Gal4 binary expression system was used to drive the expression of transgenic constructs [37]. For generation of the UAS-*raw *transgenic construct, the full-length *raw *cDNA was amplified using pNB3301 (obtained from A. Letsou) as template with a pair of primers (5'-CACCATGAAAACTGAAAGCAGCAGT-3' and 5'-GCAGCGGTCGCGGTTGTTGT-3') by PCR. The amplified DNA fragment was first cloned into a pENTR/D-TOPO vector (Invitrogen), and subsequently into a pTWF vector (The *Drosophila *Genomics Resource Center). The plasmid construct was confirmed by sequencing before germ-line transformation. The *raw*-RNA interference construct, UAS-*raw*-RNAi, was generated by amplification of a 693 bp DNA fragment using the following primers, 5'-GCTCTAGACCTGGAGCGCCAGAGTCTC-3' and 5'-GCTCTAGATGACGAAGAGCAACACTCG-3'. The amplified DNA fragment was digested with *Xba*I and cloned into the *Avr*II site of a pWIZ vector. The correct clone was used to clone the same *Xba*I-digested DNA fragment into the *Nhe*I site, as described elsewhere [38]. The orientation of the inverted DNA fragment was confirmed by restriction enzyme digestion. Flies carrying transgenic constructs were generated by *P-*element-mediated germ-line transformation procedure using *w^1118 ^*as the parental line [39].

### RT-PCR

Total RNA was purified from embryos carrying *raw*-RNAi transgene driven by *Act5C-gal4 *driver using Trizol (Invitrogen). 10 μg of total RNA was reverse transcribed using oligo(dT) primers and SuperScript reverse transcriptase (Invitrogen). PCR was performed using *raw *specific primers (5'-TACCATAAGCACCGCCAGCA-3' and 5'-ATGCGAACTGGCCGAGGATC-3') and *rps17 *primers (5'-CGAACCAAGACGGTGAAGAAG-3' and 5'-CC TGCAACTTGATGGAGATACC-3'). PCR condition: at 94°C for 2 min, 30 cycles (at 94°C for 30 sec, at 46°C for 30 sec, at 72°C for 1 min), and at 72°C for 7 min.

### Cell death detection

Apoptosis was detected by acridine orange (AO) staining or terminal deoxynucleotidyl transferase-mediated dUTP nick end labeling (TUNEL). For AO staining, the same protocol was followed as used elsewhere [40]. Briefly, dechorionated embryos were stained by placing in an equal volume of *n*-heptane and 1 × PBS containing 5 mg/ml of acridine orange (Sigma) for 5 minutes on a shaking platform. For TUNEL analysis, an in situ cell death kit was used according to the manufacturer's instructions (Roche Applied Science). To detect cardiac apoptosis in embryos, a TUNEL and immunoflourescence double-labeling protocol was followed as described [41]. Stained embryos were mounted either with mineral oil (Sigma) or series 700 Halocarbon oil (Halocarbon Products, Hackensack, NJ). Samples were viewed either with a fluorescence or TCS SP2 confocol microscope (Leica Microsystems).

### Immunohiscytochemistry, X-gal staining and cuticle preparation

Immunohistochemical staining was performed as described [42]. Antibodies and dilutions were as follows: Anti-Eve, anti-Wg 4D4 and anti-EC11 (1:10; obtained from the Developmental Studies Hybridoma Bank), anti-Tin (1:2000, provided by M. Frasch), anti-LacZ, anti-GFP (1:200, Molecular Probes), and anti-pMad (1:20, Cell Signaling) [43]. Appropriate anti-mouse or anti-rabbit HRP-conjugated secondary antibodies were used at a dilution of 1:200 (Jackson ImmunoResearch). The staining pattern was visualized using the Vectastain ABC kit (Vector Laboratories). X-gal staining was performed as described previously with slight modifications [44]. Briefly, collected embryos were dechorionated in 50% bleach for 90 s and fixed in 4% formaldehyde buffered with 1× PBS for 20 min. Fixed embryos were washed briefly and incubated with X-gal staining solution (10 mM sodium phosphate, pH 7.2, 150 mM NaCl, 1 mM MgCl_2_, 3 mM K_4_[Fe(CN)_6_], 3 mM K_3_[Fe(CN)_6_], 0.3% Triton X-100, 0.2% X-gal) at 25°C. Immuno- and X-gal stained embryos were mounted in 50% glycerol. For preparation of cuticle, embryos were fixed in glycerol-acetic acid (1:4) and cleared in Hoyer's medium overnight at 60°C [45]. Stained embryos and cuticle were visualized with a light microscope (Leica DMR A2).

### Image acquisition and processing

Epifluorescence images were acquired using a digital camera (CoolSnap 5.0, Photometrics) steered by the Northern Eclipse 6.0 software (EMPIX Imaging, Mississauga, Ontario, Canada). When necessary, Z-series of optical or fluorescent images were acquired at 2 μm increments with a piezo-electric motor (LVDT, Physik Instruments). The Helicon Focus program was applied to combine the focused images (Helicon Soft Ltd. Kharkov, Ukraine). All the figures were arranged in Adobe Illustrator CS3 (Adobe Co.).

## Results

### Loss of *raw *function impairs heart and muscle development

**Figure 1:**
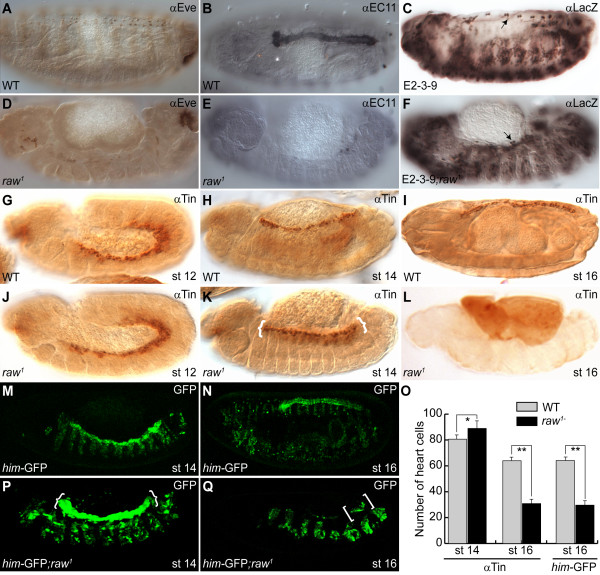
**Cardial phenotypes of *raw *mutant embryos**. (A, B, C, G, H, I, M, N) Wild-type (WT) embryos; (D, E, F, J, K, L, P, Q) *raw^1 ^*mutant embryos. (A) EPC was visualized with anti-Eve antibodies at stage 16. (D) EPC was completely absent in *raw^1 ^*mutant embryos. (B) The EC11 antibody was used to label pericardial cells at stage 16. (E) Pericardial cells were abolished in *raw^1 ^*mutant embryos. (C) E2-3-9 enhancer trap line shows the presence of 2 *svp*-specific cardioblasts per hemisegment in WT embryos (arrows). (F) Most *svp*-expressing cardioblasts were absent in *raw *mutants. (G) The expression of Tin in dorsal mesoderm was revealed using anti-Tin antibodies in WT embryos at stage 12. (J) The expression of Tin was not affected in *raw^1 ^*mutant embryos at similar stages. (H) Tin was expressed in heart cells at stage 14 in wild type embryos. (K) Overproduction of heart precursors was observed in *raw^1 ^*mutant embryos at stage 14 (braces). (I) Tin was confined in matured myocardial cells at stage 16 in the wild type (L). The number of myocardial cells was greatly reduced in *raw^1 ^*mutants during late embryogenesis (brackets). (M) Cardial and muscle cells were monitored by the expression of *him*-GFP reporter at stage 14. (P) The number of *him-*GFP expressing cardial precursors increased dramatically (braces). Note that the enhancer activity of *him *was also enhanced in musculature of *raw^1 ^*mutants at stage 14. (N) The expression of *him*-GFP was confined to all cardial and pericaridial cells, while its expression in muscle cells was reduced greatly at stage 16. (Q) The *him-*GFP expressing heart cells were dramatically reduced (brackets). Note that the expression of *him-*GFP in muscle persisted in *raw^1 ^*mutants during late embryogenesis. (O) Number of heart cells in wild-type (gray bar) and *raw^1 ^*mutant (black bar) embryos at stage 14 and 16 was revealed by Tin immunoreactivity and expression of *him-GFP *reporter. Data were expressed as the mean ± SD and were analyzed by Student's *t*-test (*n *= 15). * indicates *p <*0.01; ** indicates *p <*0.001. st, stage.

In our effort to identify genes involved in the heart development of *Drosophila*, we found that many cardial cell types were missing in *raw *mutant embryos. For instance, using anti-Eve antibodies we found that Even-skipped positive pericardial cells (EPCs) were aligned normally along the dorsal vessel in wild-type embryos (Figure 1A). However, these EPCs almost completely disappeared in the *raw *mutant at stage 16 (Figure 1D). Similarly, absence of pericardial cells was observed in *raw *mutant using anti-EC11 and anti-Odd antibodies, which labelled extracellular matrix and Odd-skipped pericardial cells (OPCs), respectively, of pericardial cells (Figure 1B, E and Additional file 1, Fig. S1). Additionally, using a heart-specific enhancer trap line, E2-3-9, we found that *svp*-expressing myocardial cells were reduced and/or missing under the *raw *mutant background (Figure 1C vs. 1F, arrow). To further investigate whether *raw *is involved in the early cardiogenesis of *Drosophila*, we used Tin as a marker because it is expressed initially in all cardial progenitors and later in four of six cardioblasts per hemisegment as well as a subset of pericardial cells in the mature embryonic heart (Figure 1H and 1I; see also [46-49]). We found that the expression of Tin in the dorsal mesoderm was normal in both wild-type and *raw *mutant embryos at stage 12 (Figure 1G and 1J). However, Tin-expressing heart cells were mildly overproduced in *raw *mutants at stage 14 (Figure 1K, braces). Nevertheless, these overproduced heart cells were reduced and/or absent in *raw *mutants during late embryogenesis (Figure 1L). Since the above markers label most of the cardial and pericardial cell types, our results suggest that the *raw *mutation affects all the cardial cell types in developing *Drosophila *heart. Based on the above observations, we thus concluded that heart cells were over-specified mindedly during mid-embryogenesis, and that these overproduced heart cells disappeared at late stages in *raw *mutant embryos.

To further confirm our findings, we used a *him*-GFP reporter in which a cardial-specific enhancer of *him *was placed upstream of nuclear GFP [33]. The GFP reporter was expressed in the precursors of both muscle and heart cells at stage 12 and its cardiac expression persisted till late embryogenesis in the differentiated heart cells under the control of *tin*, while its muscle expression was greatly reduced at stage 14 (Figure 1M and 1N; and M-T. Su unpublished results). We found that the expression of *him-*GFP was increased in both heart and musculature at stage 14 under the *raw *mutation background (Figure 1P). Consistent with the above data, cardiac expression of *him-*GFP decreased significantly in mutant embryos at stage 16 (Figure 1O and 1Q). Taken together, these data show that heart precursors are overspecified during mid-embryogenesis, but are missing in late mutant embryos.

### Down-regulation of *raw *causes cardial apoptosis

**Figure 2:**
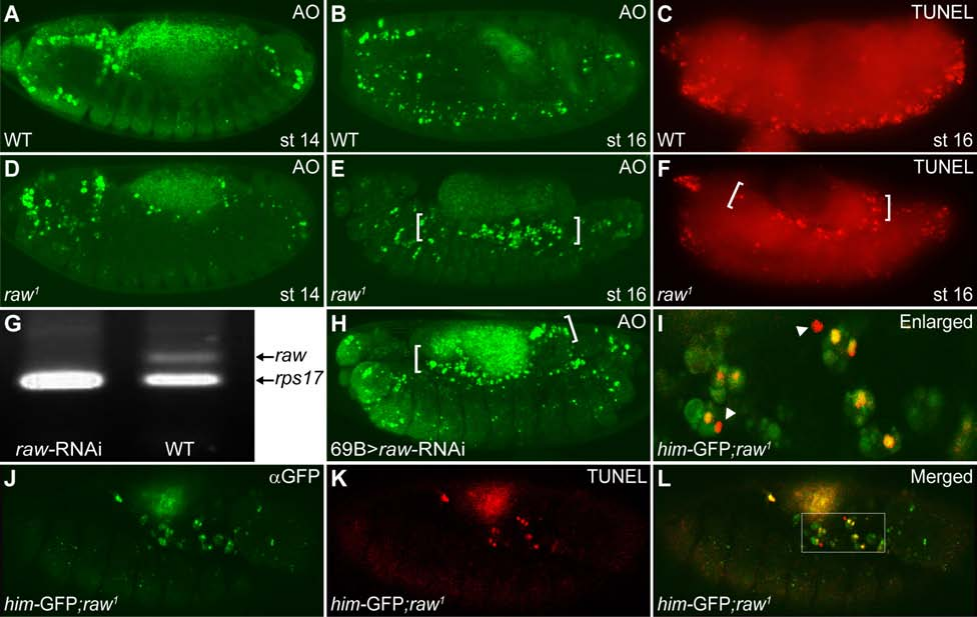
**Down-regulation of *raw *causes cardial apoptosis**. Epifluorescence micrographs showed localized cell death in the *raw *mutant. (A-C) Wild-type (WT) embryos, (D-F) *raw^1 ^*mutant embryos. (A) Acridine orange (AO) positive cells were detected mainly in cephalic ganglia and head regions at stage 14. (D) *raw^1 ^*mutant embryos of the same age displayed a similar AO staining pattern. Normal PCD detected using (B) AO or (C) TUNEL staining in wild-type embryos at stage 16. Excessive cell death (brackets) was observed in dorsal mesoderm of *raw^1 ^*mutant embryos using (E) AO or (F) TUNEL staining. (G) Real time PCR quantification of *raw *mRNA level relatively to *rps17 *mRNA. (H) Knocking down the expression of *raw *in the ectoderm caused excessive cell death (brackets) in the dorsal mesoderm. (I-L) Double-labeling of dying cells using immunostaining of (J) *him-GFP *expressing cells (green) and (K) TUNEL (red) in *raw^1 ^*mutant was assessed by confocol microscopy. (I) High magnification view of TUNEL and anti-GFP immunostained embryos from marked area (white box) in (L) merged image. Dying cells (yellow nuclei surrounded with green cytoplasma); Healthy *him*-GFP-positive cells (green); Unidentified dying cells (red, white arrowheads). WT, wild-type; st, stage.

How could the cardial cells disappear in the *raw *mutant embryos at later stages? One possibility is that loss of *raw *function induces programmed cell death (PCD). To investigate whether cardial apoptosis occurred in *raw *mutant embryos, we stained embryos with a vital dye, acridine orange (AO), which provides a rapid visual assessment of apoptosis in live *Drosophila *[40]. Epifluorescence micrography showed apoptotic cells, mainly in the cephalic ganglia and in head regions in wild-type embryos at stage 14 (Figure 2A). A similar AO staining pattern was detected in *raw^1 ^*mutant embryos at the same stage (Figure 2D). Excessive cell death in the dorsal mesoderm was observed in *raw^1 ^*mutant embryos at stage 16 (Figure 2B vs. 2E). To verify the above findings, terminal deoxynucleotidyl transferase dUTP nick end labeling (TUNEL) was applied. Indeed, TUNEL positive nuclei were identified in the dorsal mesoderm of *raw^1 ^*mutant embryos at stage 16 (Figure 2C vs. 2F). We then made use of RNA interference to specifically knock down the expression of *raw *(Figure 2G and 2H), and the results were consistent with the above findings, founding that silencing the expression of endogenous *raw *in ectoderm using *69B-gal4 *successfully induced localized apoptosis in the dorsal mesoderm of embryos (Figure 2H).

Since the embryonic heart of *Drosophila *is located in the dorsal mesoderm, which was also the region where cell death was present in the *raw *mutants, we suspected that PCD is the cause of eliminating cardial cell types in the *raw *mutant. To test this, we double-labeled *raw *mutant embryos with TUNEL and heart-specific *him*-GFP reporter (Figure 2I-L). By confocal microscopy analysis, the results showed that many *him-*GFP expressing cells were co-labeled with TUNEL, indicating that mesodermally-derived tissues did undergo apoptosis (Figure 2I, yellow nuclei surrounded with green cytoplasma). Since the expression of *him-*GFP was mainly in cardial and muscle cells under *raw *mutation background at late stages (Figure 1Q), these results indicate that *raw *mutation does induce death of heart cells. Apart from the *him-*GFP-expressing cardial and muscle cells, we noticed few unidentified TUNEL-positive cells (Figure 2I, red, arrowheads). Since heart is the major defective tissue in the mesoderm of *raw *mutant, we have focused our attention on how loss of *raw *function leads to cardial apoptosis in *Drosophila *during late embryogenesis.

### Function of *raw *is required in ectoderm

**Figure 3:**
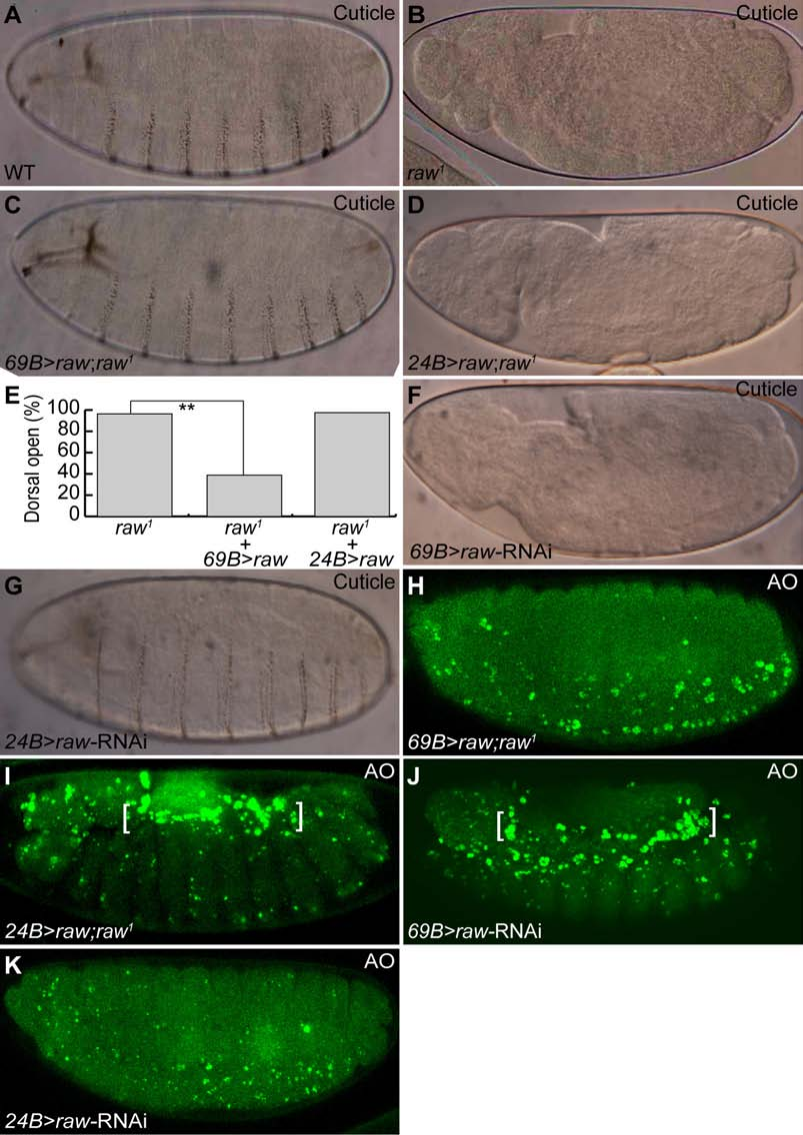
**Ectodermal *raw *is sufficient for the normal development of *Drosophila***. (A-D, F, G) Cuticular phenotype. (A) Wild type (WT). (B) *raw^1^*. (C) UAS-*raw; raw^1^*/*69B-gal4*; *raw^1^*. (D) UAS-*raw; raw^1^*/*24B-gal4*; *raw^1^*. (E) Bar chart showing that ectodermally, but not mesodermally, expressed *raw *suppressed dorsal open phenotype in *raw *mutants. Statistical analysis of the percentage of embryos with dorsal open phenotype in the indicated genetic background. Number of scored embryos for each genotype: *raw^1 ^*(*n = *544); *69B>raw; raw^1 ^*(*n = *414); *24B>raw; raw^1 ^*(*n = *324). Chi-square test, ** indicates *p <*0.001. (F) UAS-*raw*/*69B-gal*. (G) UAS-*raw-*RNAi/*24B-gal4*. (H-K) Apoptosis phenotype revealed by AO staining. Ectodermally (H), but not mesodermally (I), expressed *raw *blocked cardial apoptosis (brackets) in *raw *mutants. *raw*-RNAi transgene driven by *69B-gal4 *(J), but not *24B-gal4 *(K), caused cardial apoptosis (brackets).

Previous study showed that mutation of *raw *resulted in dorsal open phenotype [35]. Ventral denticle belts are also missing in embryos homozygous for the null *raw^1 ^*allele (Figure 3B, see also [50]). As demonstrated above, lack of *raw *function leads to apoptosis of dorsal mesodermal tissues, including heart. These findings suggest that *raw *is a pleiotropic gene which is required for the normal development of multiple tissues in *Drosophila*. To determine the spatial requirement of *raw*, we performed rescue experiments by expressing *raw *using *69B- *or *24B*-*gal4 *driver, which direct the expression of UAS-*raw *in ectoderm or mesoderm respectively (*69B*>*raw *or *24B>raw*). Consistent with a previous study, we found that the cuticular phenotypes of *raw *mutant could be rescued by targeted expression of Raw protein under the control of *69B-gal4 *(Figure 3A, C, E, see also [50]). In fact, 55% of the *raw *mutant flies survived to adulthood after rescue of the cuticular phenotype (data not shown). By contrast, expression of the UAS-*raw *transgene driven by *24B*-*gal4 *failed to restore the dorsal open or the loss of denticle belt phenotypes (Figure 3D and 3E). Conversely, we were able to replicate the cuticular phenotype by knocking down endogenous *raw *using a *69B-gal4 *driver (Figure 3F). However, no cuticle defect was observed when the *raw*-RNAi was driven with the pan-mesoderm driver *24B*-*gal4 *(Figure 3G).

Rescue experiments were also conducted to determine whether *raw *functions cell-autonomously for the survival of mesodermally-derived tissues. We found that epidermal expression of *raw *suppressed cardial apoptosis under *raw *mutant background (Figure 3H). However, mesodermally-overexpressed *raw *was unable to completely inhibit cardial apoptosis in the *raw *mutant (Figure 3I, brackets). Additionally, transgenic *raw*-RNAi construct driven by *69B-gal4*, but not *24B-gal4*, produced a cardial apoptosis phenotype (Figures 2H, 3J vs. Figure 3K, brackets). These results strongly suggest that ectodermal Raw is sufficient for the proper development of *Drosophila*.

### Raw affects Dpp and Wg signalings

**Figure 4:**
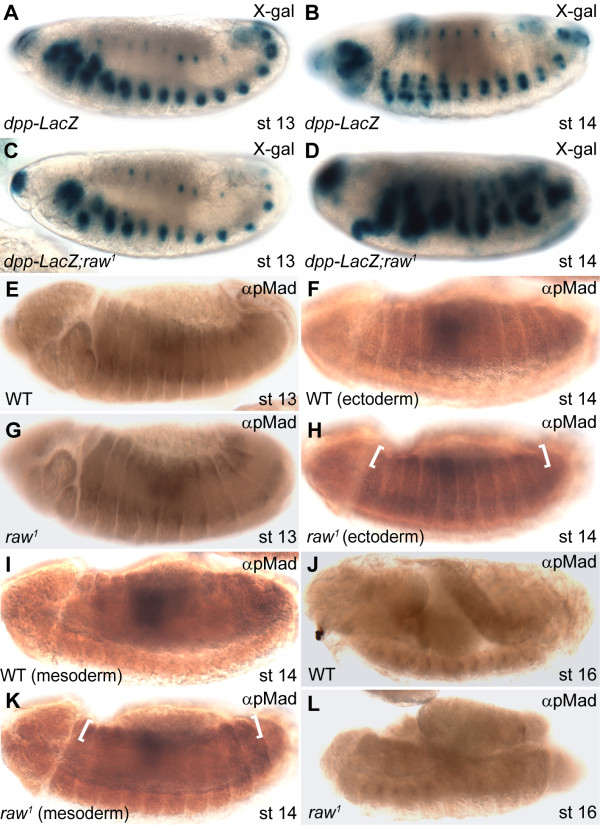
**Dpp signalings was affected in *raw *mutants**. (A, C) The expression of *dpp*-LacZ was normal in both wild-type and *raw *mutants at stage 13. (B, D) At stage 14, *dpp*-LacZ was expressed in the leading edge of wild-type embryos whereas *dpp*-LacZ was ectopically expressed in dorsal ectoderm of *raw *mutant embryos. (E, G) At stage 13, pMad was detected at the dorsal ectoderm in both wild-type (WT) and *raw *mutants. (F, H) Expression of pMad in epidermis was reduced in the wild-type embryos but was greatly increased in *raw *mutant embryos at stage 14 (brackets). (I, K) Optical section through the stained embryos revealed that the broad-band expression pattern of pMad was persisted in the dorsal mesoderm of *raw *mutant embryos but not in wild-type embryos. (J, L) At stage 16 pMad was detected in developing midgut and hindgut in wild-type embryos. Its expression in *raw *mutant embryos was mostly abolished. WT, wild-type; st, stage.

Having established that ectodermal Raw functions in a cell non-autonomous manner to affect the viability of dorsal mesodemal cells, we speculated that ectodermally-secreted factors might be responsible for the cardial apoptosis observed in the *raw *mutant. In this regard, Wg and Dpp are good candidates because they are essential for patterning dorsal mesodermally-derived tissues, including cardial progenitor cells. Previous study has shown that Dpp is ectopically expressed at the dorsal epidermis of *raw *mutant embryos [35]. Using a *dpp*-LacZ reporter, we confirm that Dpp signaling is ectopically activated in *raw *mutant embryos at stage 14 (Figure 4A, B vs. Figure 4C, D). To further determine if Dpp signaling is altered in *raw *mutants, we conducted immunocytochemistry experiments using a monoclonal antibody against pMad [43], a Dpp-activated Smad protein. pMad immunoreactivity was detected as a broad band in the dorsal ectoderm of both wild-type and *raw *mutant embryos at stage 13 (Figure 4E and 4G). The broad band expression pattern disappeared in wild-type embryos but not in the dorsal ectoderm of *raw *mutants at stage 14 (Figure 4F vs. 4H, brackets). Moreover, optical sectioning through the stained embryo showed the same broad band expression pattern of pMad in mutant but not in wild-type embryos (Figure 4I and 4K, brackets). At late stages, pMad was not detected in the dorsal ectoderm of either mutant or wild-type embryos (Figure 4J and 4L).

Immunostaining was also used to investigate if Wg signaling is affected by the *raw *mutation. We found that Wg was expressed in transverse striped domains of the ectoderm along the anteroposterior axis in wild type embryos at stages 13 (Additional File 2, Fig. S2). The expression pattern of Wg was not altered in *raw *mutants by stage 13. However, the expression level of Wg had significantly decreased in *raw *mutants at stage 14 (Additional File 2, Fig. S2). At stage 16, residual Wg was detected in the ectoderm of wild-type embryos, but was completely absent in *raw *mutant embryos (Additional File 2, Fig. S2).

### Ectopic Dpp signaling promotes cell death

To investigate if down-regulation of Wg signaling causes cardial apoptosis, we conducted a temperature shift experiment using a temperature-sensitive *wg^IL114 ^*allele. Inactivation of Wg at 9-15 hr, the time window during which Wg expression is missing in *raw *mutant embryos, did not cause the cardial apoptosis seen in *raw *mutant embryos, and overexpression of *wg *did not suppress the apoptosis phenotype under *raw *mutation background (Additional File 2, Fig. S2). We thus concluded that defective Wg signaling is not the cause of cardial apoptosis.

**Figure 5:**
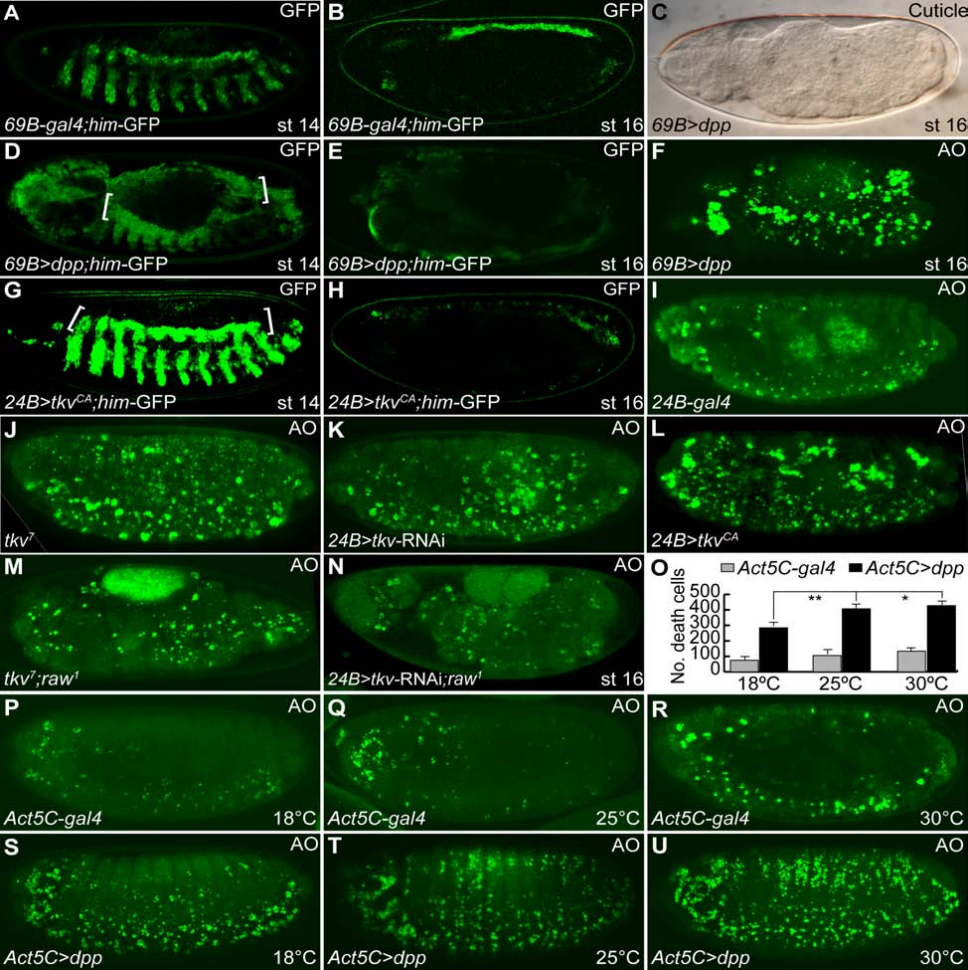
**Ectopic Dpp signaling induces apoptosis**. (A) *him*-GFP reporter is expressed in muscle and heart precursors in control *69B-gal4 *drivers at stage 14. (B) The expression of *him-*GFP reporter was confined to cardial and pericardial cells at stage 16. (C-F) Overexpression of *dpp *in ectoderm using *69B-gal4 *driver mimicked the *raw *mutant phenotype. (C) Ectodermal expression of *dpp *resulted in naked cuticle with dorsal open phenotype. (D) Cardiac *him-*GFP was overexpressed in embryos overexpressing *dpp *at stage 14 (brackets). (E) Cardial and muscular *him-*GFP was lost in embryos overexpressing *dpp *at stage 16. (F) Ectopic *dpp *induced excessive cell death. (G) Overexpression of *tkv^CA ^*using *24B*-*gal4 *increased the expression of *him-*GFP reporter at stage 14. (H) The expression of *him-*GFP was abolished in embryos overexpressing *tkv^CA ^*at stage 16. (I) Normal developmental PCD revealed by AO staining in control *24B-gal4 *driver. (J) AO-positive cells were moderately increased in *tkv^7 ^*mutant embryos. (K) RNAi silenced *tkv *under the control of *24B-gal4 *induced apoptosis. (L) Mesodermal overexpression of *tkv^CA ^*caused excessive cell death. (M) Scattered AO-positive cells were present in *tkv^7^; raw^1 ^*double mutant embryos. Note that the mutant embryos did not exhibit cardial apoptosis, but showed a dorsal open phenotype. (N) Mesodermal knockdown of the expression of *tkv *suppressed cardial apoptosis in *raw *mutant. (O-U) Ectopic *dpp*-induced apoptosis was dose-dependent. (P-R) Control *Act5C-gal4 *driver showed normal AO staining pattern at different temperatures: (P, S) 18°C; (Q, T) 25°C; (R, U) 30°C. (S-U) Constitutive overexpression of *dpp *induced apoptosis. (O) Dead cells were quantified and expressed as the mean ± SD values. Comparison of dead cells among three groups was assessed by one-way analysis of variance (ANOVA) followed by Dunnett's post-hoc test (*n = *15). * indicates *p <*0.05; ** indicates *p <*0.001. WT, wild-type; st, stage.

We then turned our attention to the question of whether ectopic Dpp signaling can induce cardial apoptosis, and found that overexpression of *dpp*, using *69B-gal4 *(*69B>dpp*), phenocopied the *raw *mutant phenotypes (Figure 5A-F). For instance, *him*-GFP expressing heart cells were overproduced at stage 14, but they had disappeared in *69B>dpp *embryos at stage 16 (Figure 5A-B and Figure 5D-E). Naked cuticle and dorsal open phenotypes were also observed in *69B>dpp *embryos (Figure 5C). However, unlike *raw *mutant embryos in which only *him*-GFP expressing cardioblasts were lost, both heart and muscle cells were completely abolished in *69B>dpp *embryos at stage 16 (Figure 1Q and 5E). This loss of *him*-GFP-expressing myoblasts and cardioblasts in *69B>dpp *embryos is very likely to have resulted from apoptosis, because extensive cell death was detected throughout the entire *69B>dpp *embryos, whereas dead cells were located mainly in the dorsal mesoderm in *raw *mutants (Figure 2E and 5F). The difference in the apoptosis phenotype between *raw *mutant and *69B>dpp *embryos might reflect the fact that the ectopic *dpp *was mainly found in the dorsal ectoderm of *raw *mutant whereas *dpp *was expressed in the entire ectoderm under the control of *69B-gal4*. These findings reinforce our hypothesis that Dpp activity is correlated with cardial apoptosis.

Dpp is a secretory protein which can be transduced to the underlying mesoderm. If the apoptotic phenotype is associated with the ectopic Dpp activity, it would expect that mesodermal overexpression of Dpp should replicate the apoptosis phenotypes seen in *69B>dpp *embryos. Indeed, mis-expression of *dpp *in mesoderm using a pan mesodermal driver, *24B-gal4*, induced excessive cell death accompanied with elimination of *him*-GFP-expressing cells during late developmental stages (Additional File 3, Fig. S3). These results suggest that excessive *dpp *in ectoderm or mesoderm can cause apoptosis and removal of cardial cells.

Dpp transduces its signaling by binding to a heteromeric type I/type II transmembrane serine/threonine kinase receptor complex, encoded by *tkv *and *punt*. To further demonstrate that ectopic Dpp signaling is the cause of apoptosis in *raw *mutants, we targeted overexpression of a constitutively-active form of *tkv*, *tkv^CA^*, using a *24B-gal4 *driver (*24B> tkv^CA^*). We found that *24B>tkv^CA ^*embryos exhibited the same phenotype as *69B>dpp *or *24B> dpp *embryos, in which *him*-GFP-expressing cardial cells were increased in *24B> tkv^CA ^*embryos at stage 14 (Figure 5G), while *him*-GFP-expressing heart and muscle cells were eliminated at stage 16 (Figure 5H). Extensive cell death was also detected throughout *24B> tkv^CA ^*embryos (Figure 5L).

In a complementary experiment, we depleted the expression of *tkv *by RNAi using a transgenic fly carrying inverted repeats corresponding to *tkv*, under the control of a UAS sequence inducible by *24B-gal4 *(*24B>tkv*-RNAi, Additional file 4, Fig. S4). Down-regulation of mesodermal *tkv *induced a moderate degree of apoptosis in which the AO-positive cells were randomly distributed in the entire *24B>tkv*-RNAi embryos (Figure 5I vs. 5K). A similar pattern of scattered cell death was also detected in loss-of-function of *tkv *mutant embryos (Figure 5J). These data suggest that Dpp is required for cell survival during embryogenesis in *Drosophila*. To test whether inhibiting Tkv activity can block excessive Dpp-induced apoptosis, we compared the AO staining pattern in *raw*, *tkv *and *raw;tkv *double mutant embryos. Both *tkv *and *raw;tkv *double mutant embryos exhibited the same scattered AO staining pattern, unlike the cardial apoptosis phenotype observed in *raw *mutant (Figure 2E vs. 5J and 5M). Moreover, mesodermal knockdown of the expression of *tkv *was able to suppress cardial apoptosis under the *raw *mutant background (Figure 5N). These results indicate that ectopic Dpp induced apoptosis is tranduced through Tkv.

As above-mentioned, ectopic *tkv *expression promoted apoptosis in *24B>tkv^CA ^*embryos, indicating that Dpp can function as a pro-apoptotic signaling. To directly test this hypothesis, Dpp was constitutively overexpressed under the control of the ubiquitous *Act5C-gal4 *driver (*Act5C>dpp*). In control *Act5C-gal4 *embryos, AO staining revealed that apoptotic cells were mainly present in the ventral nerve cord and head regions at stage 16 (Figure 5P-R). Raising the temperature slightly increased the amount of cell death in the central nervous system of late *Act5C-gal4 *embryos (Figure 5O-R). Constitutive overexpression of Dpp caused a remarkable degree of apoptosis in *Act5C>dpp *embryos (Figure 5S-U). Notably, increasing the *dpp *expression level by raising the temperature significantly increased the number of dying cells (Figure 5O-U). This result suggests that Dpp can function as a pro-apoptotic signaling in a dose-dependent manner.

### Dpp induced apoptosis is mediated through DTAK1

**Figure 6:**
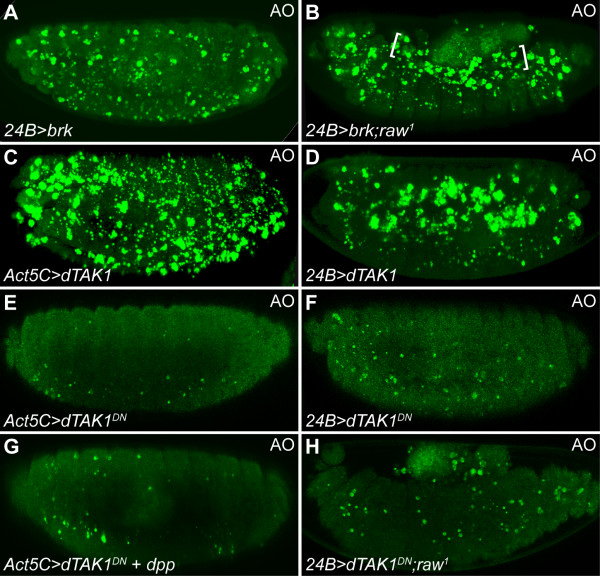
**Ectopic Dpp-induced apoptosis is mediated through DTAK1**. (A) Ectopic expression of *brk *using *24B-gal4 *induced cell death. (B) Mesodermal overexpression of *brk *did not suppress cardial apoptosis in *raw *mutants (brackets). (C, D) Excessive cell death was induced by overexpression of *dTAK1 *using *Act5C- *or *24B-gal4 *drivers. (E, F) Overexpression of dominant negative *dTAK1^DN ^*using *Act5C- *or *24B-gal4 *drivers inhibited apoptosis. (G) Constitutively-expressed *dTAK1^DN ^*suppressed ectopic *dpp*-induced apoptosis. (H) Mesodermal expression of *dTAK1^DN ^*inhibited cardial apoptosis in *raw *mutants.

As shown above, reducing Tkv activity successfully blocked *dpp*-mediated cardial apoptosis in *raw *mutants (Figure 5M and 5N). This data encouraged us to further examine whether overexpression of *brinker *(*brk*), a transcriptional repressor of Dpp, could inhibit the cardial apoptosis phenotype in *raw *mutants. Overexpression of *brk *using *24B-gal4 *driver (*24B>brk*) caused a moderate degree of apoptosis (Figure 6A). The AO staining pattern in *24B>brk *embryos was similar to the pattern in *tkv^7^*and *24B>tkv-*RNAi embryos (Figures 6A, 5J, and 5K), suggesting that ectopic *brk*-induced apoptosis was likely to be a result of the inhibition of Dpp signaling. Unexpectedly, mis-expression of *brk *in mesoderm failed to suppress the cardial apoptosis phenotype of the *raw *mutant (Figure 6B, brackets), suggesting that the suppressor activity of Brk may not be strong enough to block Dpp-mediated cardial apoptosis. Nevertheless, it is equally possible that Dpp-induced apoptosis is mediated through a distinct pathway which can not be repressed by Brk.

A previous study showed that TGF-β activated kinase 1 (TAK1), a member of the JNKK kinase superfamily that activates the JNK cascade, can transduce TGF-β signaling and induce apoptosis in vertebrates [51]. To determine if TAK1 induces apoptosis in response to Dpp signaling, a *Drosophila *TAK1 homolog, DTAK1, was mis-expressed using either *Act5C- *or *24B-gal4*. Ectopic DTAK1 activity was sufficient to induce apoptosis in embryos (Figure 6C and 6D). In contrast, ectodermal or mesodermal overexpression of a dominant negative form of *dTAK1 *(*dTAk1^DN^*) was capable of suppressing developmental programmed cell death (Figure 6E and 6F). Constitutive overexpression of *dTAK1^DN ^*also effectively inhibited ectopic Dpp-induced apoptosis (Figure 6G). Moreover, mesodermally overexpressed *dTAK1^DN ^*suppressed cardial apoptosis under the *raw *mutation background (Figure 6H). Taken together, our results clearly demonstrate that DTAK1 is a downstream effector of Dpp-mediated cardial apoptosis.

### DTAK1 induces JNK dependent apoptosis

**Figure 7:**
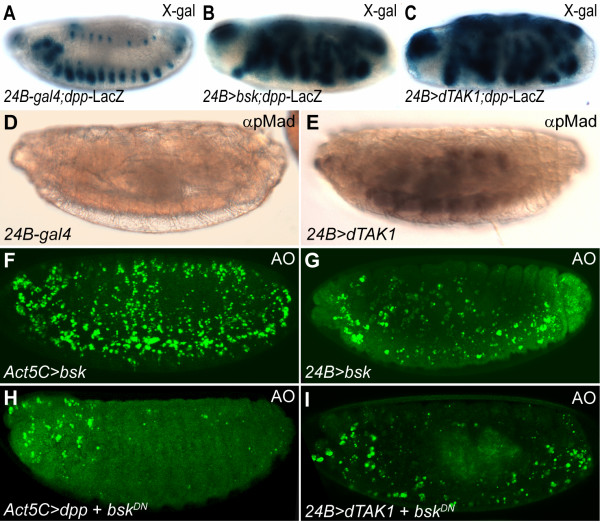
**DTAK1 activates DJNK pathway**. (A) *dpp-*LacZ showed a typical expression pattern in dorsal and lateral spots along the anterior-posterior axis in control *24B-gal4 *driver embryos. (B) Mesodermal *bsk *induced the expression of *dpp*-LacZ. (C) *dpp*-LacZ reporter was ectopically expressed in *24B>dTAK1 *embryos. (D) pMad was not ectopically expressed in control *24B-gal4 *driver. (E) Targeted overexpression of *dTAK1 *in mesoderm induced ectopic pMad. (F, G) Excessive apoptosis was induced by the expression of *bsk *using *Act5C- *or *24B-gal4 *drivers. (H) Expression of *bsk^DN ^*inhibited ectopic *dpp*-induced apoptosis. (I) Mesodermal expression of *bsk^DN ^*inhibited *dTAK1*-induced cell death.

Although our data suggest that *dTAK1 *acts downstream of *dpp *to promote apoptosis, it is interesting to note that *dTAK1 *stimulates *dpp *expression, suggesting that *dpp *is a downstream target of *dTAK1 *[29]. Despite the discrepancy in these epistatic relationships, these observations strongly suggest that *dTAK1 *and *dpp *act in the same genetic pathway. Using a *dpp-*LacZ reporter, we found that *dTAK1 *activated the transcription of *dpp *to the same degree as *basket *(*bsk*), the *Drosophila *Jun amino-terminal kinase (DJNK) homolog encoded gene which activates the expression of *dpp *in the dorsal-most epidermal cells (Figure 7A-C). These results indicate that *dTAK1 *acts upstream of *dpp*. Since *dpp *is also activated by *bsk*, these results suggest that *dTAK1 *activates *bsk *and thereby *dpp*. Moreover, we found that overexpression of *dTAK1 *using *24B-gal4 *driver stimulated the expression of pMad in the entire mesoderm (Figure 7D vs. Figure 7E), indicating that the ectopic Dpp induced by DTAK1 can transduce its own signaling through Tkv and result in the expression of ectopic pMad. As Dpp can transduce its signaling through DTAK1 to promote apoptosis (Figure 6G), the ectopic Dpp induced by DTAK1 is likely to autoregulate itself through DJNK. In other words, a positive autoregulatory loop for Dpp expression is formed when DTAK1 is activated.

The JNK cascade is a well-known pro-apoptotic signaling that participates in stress-related apoptosis in *Drosophila*. Together with the fact that TAK1 can activate the JNK cascade, the autocrine Dpp is expected to enhance the activity of DTAK1 and thereby hyperactivitates the DJNK pathway. In other words, the ectopic *dpp*-induced cell death could be a consequence of hyperactivated JNK signaling. To test this hypothesis, *bsk *was overexpressed in the entire embryo or specifically in mesoderm using either *Act5C- *or *24B-gal4 *driver (*Act5C>bsk*; *24B>bsk*). Ectopic *bsk *activity dramatically increased levels of apoptosis in the embryos (Figure 7F and 7G). Conversely, overexpression of a dominant negative *bsk*, *bsk^DN^*, again using either *Act5C- *or *24B-gal4 *driver, was able to suppress ectopic Dpp/DTAK1-induced apoptosis (Figure 7H and 7I). These results demonstrate that Dpp exerts its pro-apoptotic functions through DTAK1 as well as through DJNK.

### Overexpression of anti-apoptotic P35 or dominant negative P53 blocks Dpp induced apoptosis

**Figure 8:**
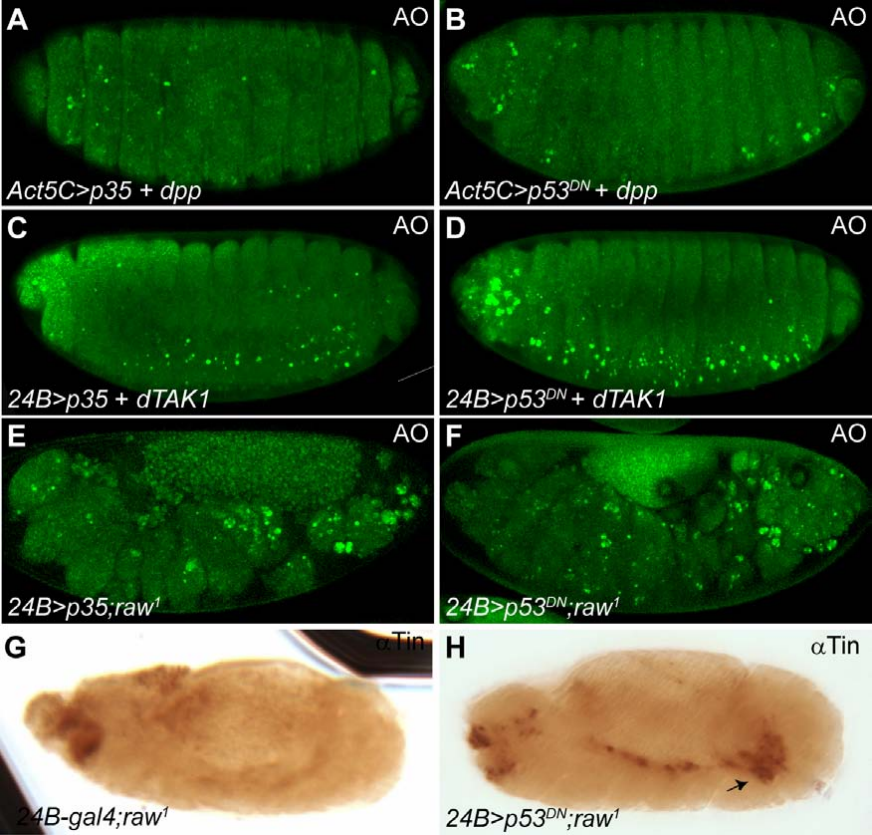
**JNK induced cardiac apoptosis is *p53 *dependent**. (A, B) Constitutive overexpression of *p35 *or dominant negative *p53^DN^*, using *Act5C-gal4 *driver, antagonized *dpp*-induced apoptosis. (C, D) Mesodermal expression of *p35 *or *p53^DN ^*suppressed *dTAK1-*induced apoptosis. (E, F) Overexpressed *p35 *or *p53^DN ^*driven by *24B-gal4 *suppressed apoptosis in *raw *mutants. (G) Loss of *raw *function resulted in loss of heart cells as revealed by anti-Tin antibodies in control *24B-gal4 *driver. (H) Excessive Tin-positive heart cells were present when *p53^DN ^*was expressed in *raw *mutants (arrow).

As demonstrated above the missing of cardial cells in *raw *mutants is likely to be a consequence of excessive *dpp *induced apoptosis. If this is the case, we expect that blocking cell death would reverse the cardial apoptosis phenotype in *raw *mutant embryos. The baculovirus P35 protein has been shown to suppress normal and induced apoptosis by inhibiting caspases in animals [36]. We found that overexpression of the anti-apoptotic P35 could prevent apoptotic cell death induced by either ectopic Dpp or DTAK1 (Figure 8A and 8C). P35 also significantly reduced the death of heart cells under the *raw *mutation background (Figure 8E). Moreover, it has been shown that aberrant JNK-induced apoptosis is mediated through P53 (reviewed in [52]). To further demonstrate if Dpp/DTAK1-induced apoptosis is P53-dependent, a dominant negative form of *p53 *(*p53^DN^*) was ectopically expressed using either *Act5C- *or *24B- gal4 *driver (*Act5C>p53^DN ^*or *24B> p53^DN^*). We found that overexpresion of *p53^DN ^*suppressed Dpp- or DTAK1-induced apoptosis successfully (Figure 8B and 8D), suggesting that Dpp/DTAK1-induced apoptosis is mediated through P53. We also showed that mesodermal overexpression of P53^DN ^significantly reduced the death of heart cells in *raw *mutant embryos (Figure 8F). Consistent with the above observation, immunostaining experiments revealed that mesodermally-overexpressed P53^DN ^was able to rescue Tin-positive cardial cells in *raw *mutant embryos (Figure 8G and 8H). In fact, superfluous Tin-positive cells were usually observed when cardial apoptosis was blocked by ectopic P53^DN ^expression (Figure 1I vs. Figure 8H, arrow), suggesting that the overspecified cardial cells during mid-embryogenesis in *raw *mutants survived when apoptosis was blocked. These results also indicate that the Dpp-DTAK1-DJNK mediated apoptosis pathway is likely to be P53-dependent.

## Discussion

### Function of Raw in DJNK signaling pathway

Analysis of the amino acid sequence of Raw revealed that it does not comprise any specific functional domain or motifs. Despite the fact that the C-terminus of Raw protein is rich in glutamine residues, a characteristic feature of some transcription factors, Raw protein was mainly detected in cytoplasma, suggesting that it may not play a role in transcriptional regulation. The most prominent feature of Raw protein is that it contains two duplicated domains with a 32 amino acid core repeat (designated as Raw repeats) that is highly conserved in invertebrates [35]. The structure of Raw is currently unavailable, so no further information regarding the function of Raw can be deduced through sequence analysis.

Epistasis analysis has demonstrated that *raw *negatively regulates *jra *activity in parallel to DJNK signaling in the epidermis of *Drosophila *[35,50]. As demonstrated above, Dpp-induced apoptosis is mediated through activation of DJNK signaling (Figure 7H). If *raw *can negatively regulate DJNK, it would be expected that forced expression of *raw *would inhibit the DJNK cascade and reverse the apoptotic phenotype in the *raw *mutant. Ectodermal overexpression of *raw *did rescue the cardial apoptosis in *raw *mutants (Figure 3H). Nevertheless, targeted expression of *raw *in the mesoderm was unable to prevent cell death under the *raw *mutation background (Figure 3I). Why is the effect of mesodermal Raw so different from that of ectodermal Raw? As demonstrated above, Dpp can trigger its own expression in the mesoderm through DTAK1 and DJNK (Figure 7A-E). This auto-regulated Dpp is expected to further enhance DJNK activity, which may out-compete the suppressive activity of Raw. This might explain why mesodermal expression of *raw *was unable to suppress *raw *mutation-induced apoptosis. However, if the initial Dpp signaling in ectoderm is suppressed then it will not initiate the autocrinal Dpp signaling in mesoderm and result in cell death. Consistent with our notion, we found that ectodermally-overexpressed *raw *suppressed the expression of pMad in *raw *mutants. In contrast, targeted expression of *raw *in mesoderm did not inhibit the expression of pMad under the *raw *mutation background (Additional File 5, Fig. S5).

### Dpp functions as survival and pro-apoptotic signals

The morphogenetic function of Dpp patterns cell fates across the developing field by forming a gradient which provides position identity for the receiving cells. Similarly, it seems that Dpp controls the viability of cells in the same concentration-dependent manner. Mutant cells deprived of Dpp signaling are lost from wing disc epithelium due to DJNK activation and apoptosis [30,53]. These observations have suggested that Dpp functions as a survival factor by preventing activation of the DJNK-dependent apoptotic pathway. Additionally, down-regulation of *mad *activated JNK and caspase-3, indicating that Dpp functions as a survival factor mediated through Mad [54]. Consistent with these findings, we found that down-regulation of *tkv *or overexpression of *brk *induced moderate cell death in embryos (Figure 5J, K and 6A). Nevertheless, Dpp seems to act as a double-edged sword because increased Dpp signaling induces DJNK-mediated apoptosis in the proximal wing [30]. Similarly, in this study, we show that ectopic *dpp *or *tkv^CA ^*expression promotes DJNK-mediated apoptosis in embryos (Figure 5F and 5L). Moreover, the apoptotic propensity of Dpp is proportional to its own expression level (Figure 5O and 5S-U). Taken together, these results suggest that Dpp can act as both survival and death signals and thus, an appropriate expression level of Dpp is indispensible for the survival of cells during development.

### TAK1 is a key transducer that mediates ectopic Dpp induced apoptosis

TAK1 is also a member of the MAPKKK family, which was originally identified as a mediator of TGF-β signaling pathway [55]. It has also shown to regulate a great variety of cellular processes through activating many down-stream kinase cascades, including I-kappa B kinase complex (IKK), p38 MAPK, JNK, and AMP-activated protein kinase (AMPK) (reviewed in [56]). Unlike the canonical pathway in which members of TGF-β family elicit phosphorylation of Smad proteins, activation of TAK1 was found to function in a receptor kinase-independent manner [26]. The existence of the non-canonical pathway may explain why mesodermal overexpression of *brk *was unable to block cardial apoptosis in *raw *mutants (Figure 6B), because it seems that *brk *only suppresses Dpp target genes containing Mad consensus binding sites [57]. Interestingly, mesodermal pMad was increased in *raw *mutant or in embryos overexpressing *dTAK1 *(Figure 4K, and 7E), indicating that both canonical and non-canonical pathways were activated simultaneously in response to ectopic Dpp signaling. The fact that apoptotic cell numbers increased dramatically in *raw *mutants or in embryos overexpressing *dTAK1 *(Figure 2E, F, 6C and 6D) suggests that the non-canonical pathway suppresses the canonical pathway when cells are exposed to excessive Dpp levels. Together with the fact that the apoptotic propensity of Dpp is dose-dependent (Figure 5O), these data imply that higher levels of Dpp elicit stronger DTAK1 activity and result in apoptosis. In support of this argument, homozygous *raw *mutant animals had significant apoptosis in the dorsal-most tissues, such as the heart, where Dpp activity is at its peak (Figure 2E and 2F), and global overexpression of Dpp signaling led to ubiquitous cell death (Figure 5F, L and 5S-U).

### Late Dpp signaling in heart development

Compared to the early function of Dpp in patterning cardiogenic mesoderm, the function of Dpp during late cardiogenesis is less explored. Previously, studies had showed that numbers of various pericardial cell types, but not cardial cells, were increased in fly embryos with the *dpp^d6 ^*mutant allele, whose expression was not maintained in the dorsal ectoderm during germ-band retraction [23,24]. The expression of the mitosis marker phospho-histone 3 was concomitantly increased in the *dpp^d6 ^*mutant, suggesting that Dpp restricts the proliferation of pericardial cells specifically during late cardiogenesis [24]. As shown above, *dpp *signaling is ectopically-activated at stages 14-15 in *raw *mutants (Figure 4D, E and 4K, see also [58]); this also gives us a good opportunity to decipher late Dpp function in the developing heart of *Drosophila*. As demonstrated above, ectopic Dpp signaling leads to both overproliferation of cardial cells during mid-embryogenesis and cardial apoptosis during late embryogenesis because overexpression of *dpp *or *tkv^CA ^*replicated the *raw *mutant phenotypes (Figure 5A-H). Interestingly, both cardial and pericardial cells were eliminated in either *raw *mutant, *69B>dpp *or *24B>tkv^CA ^*embryos (Figure 1D-F, L, O, Q, 5E and Figure 5H). Our data contradict the observation that targeted overexpression of *tkv^CA ^*under the control of cardioblast-specific *tinCΔ4-gal4 *did not reduce the number of cardial cells significantly [24]. The disparate results may be due to differences in the temporospatial expression of the transgene driven by different *gal4 *drivers. Alternately, if the pro-apoptotic propensity of Dpp is concentration-dependent (Figure 5O and 5S-U), as discussed above, it is also possible that the expression of *tkv^CA ^*driven by *tinCΔ4-gal4 *is not strong enough to trigger the apoptosis response in cardioblasts.

### Model for *raw *mutation mediated apoptosis

**Figure 9:**
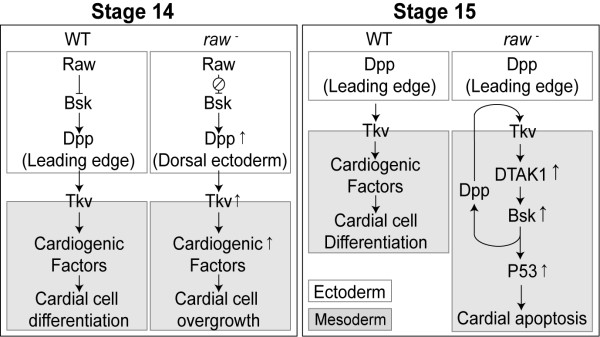
**The genetic network that leads to cardiac apoptosis in *raw *mutation**. At stage 14, Raw restricts *bsk *which limits the expression of *dpp *at ectodermal leading edge cells. Minimal Dpp activity maintains the activation of cardiogenic factors which are essential for the differentiation of cardial cells. In *raw *mutation, Bsk is de-regulated which causes overexpression of *dpp *in dorsal ectoderm at stage 14. Ectopic *dpp *signaling increases the expression of cardiogenic factors which results in overgrowth of cardial cell types at stage 14. At stage 15, while the expression of Dpp in the LE cells gradually decreases, heart cells are continuously differentiated with the function of cardiogenic factors. At stage 15, DTAK1 is activated by the ectopic Dpp signaling in *raw *mutant. DTAK1 activates DJNK and thereby Dpp. Autocrine Dpp further enhances DJNK and eventually leads to P53-dependent apoptosis.

Based on our data, we propose a model to depict the genetic pathways involved in *raw *mutation-mediated apoptosis (Figure 9). At stage 14, Dpp activity in leading edge cells activates cardiogenic factors in the underlying mesoderm which are essential for the differentiation of dorsal mesodermally derived tissues, including the heart. Deficits in Raw function cause overexpression of *dpp *which increases the activities of cardiogenic factors and results in overgrowth of cardial cells at stage 14. At stage 15, the expression of Dpp in the LE cells is maintained by the DJNK cascade, and heart cells are continuously differentiated by the function of cardiogenic factors in wild-type embryos. In *raw *mutant embryos, ectopic Dpp activates DTAK1 which triggers the expression of Bsk as well as Dpp. The induced Dpp functions in an autocrine manner to further enhance activity of Bsk and eventually lead to P53-mediated apoptosis.

In support of our model, it has been found that TGF-β/SMAD signaling exerts its apoptotic function in an autocrine loop manner in rat cardiomyocytes [59]. Induction of cardiomyocyte apoptosis by caspase overexpression has been shown to cause lethality and dilated cardiomyopathy in mice [60]. In contrast, inhibition of cardiomyocyte apoptosis by treating with a caspase inhibitor reduced apoptosis, improved cardiac function, and delayed progression of heart failure in a cardiomyopathy animal model [61]. Prolong haemodynamic overload can up-regulate TGF-β [62,63], and death of cardiomyocytes is observed in virtually every myocardial disease (reviewed in [64]. The pathway unraveled in this study is the first report that links ectopic Dpp and DJNK-dependent cardial apoptosis through the non-canonical pathway and dTAK1 activation. Our findings may thus suggest possible medical interventions for human cardiomyopathy.

## Conclusion

By analyzing the heart defect phenotype of *raw *mutant embryos, we demonstrate that overexpression of *dpp *lead to cardial apoptosis during late embryogenesis of *Drosophila*. We also demonstrate that Dpp induces its own expression through dTAK1. The activation of dTAK1 causes the hyperactivation of *Drosophila *JNK (DJNK) thereby cardial apoptosis. This is the first report that links ectopic Dpp and DJNK-dependent cardial apoptosis through the non-canonical Dpp signaling pathway and dTAK1 activation. Since haemodynamic overload usually up-regulates TGF-β, a mammalian homolog of Dpp, and death of cardiomyocytes, the pathway delineated in this study may suggest possible medical interventions for human cardiomyopathy.

## Competing interests

The authors declare that they have no competing interests.

## Authors' contributions

SAY conducted all the experiments. MTS designed the experiments and wrote the manuscript. All authors read and approved the final manuscript

## Supplementary Material

Additional file 1 **Fig. S1**. Odd-skipped pericardial cells (OPCs) are missing in *raw^1 ^*mutant embryos at late stages. (A) wild-type embryos show the presence of OPCs. (B) OPCs are completely absent in *raw *mutants. Click here for file

Additional file 2 **Fig. S2**. Loss-of Wg function does not lead to localized apoptosis. (A, D) Wg was expressed in a series of ectodermal cells at dorsal and ventral sites of embryos at stage 13. The expression pattern was not altered in *raw *mutant. (B, E). Lateral expression of Wg became a transverse stripe in the dorsal ectoderm of wild-type embryos. However, its expression decreased significantly in *raw *mutant embryos at stage 14. (C, F) At stage 16, residual Wg staining was detected in the dorsal epidermis of wild-type embryos, but its expression was completely lost in *raw *mutants at stage 16. (G) *raw *mutation shows cadial apoptosis phenotype (brackets). (H) *wg^IL114 ^*is a temperature-sensitive allele that mimics the null *wg *allele at non-permissive temperatures. Removal of *wg *function does not lead to cardial apoptosis phenotype in temperature shift experiment using *wg^IL114 ^*allele. Incubation times (9-15 hr) were normalized to development at 25°C. (I) Ectopic *wg *expression driven by *69B-gal4*, did not suppress cardial apoptosis in *raw *mutants (brackets). Click here for file

Additional file 3 **Fig. S3**. Mesodermally overexpression of Dpp induces *raw*-like phenotypes. (A,) *him-GFP *reporter was expressed in muscle and heart precursors in *24B-gal4 *control flies at stage 14. (B) Expression of *him-GFP *was limited in heart cells in control 24B-*gal4 *driver at stage 16. (C) Mesodermal overexpression of *dpp *induced ectopic heart cells at stage 14. (D) *him-GFP *expressing heart cells were lost in embryos expressing *dpp *using *24B-gal4 *at stage 16. (E, F) Normal AO staining pattern was observed in *24B-gal4 *control driver at stage 14 and 16. (G) Mesodermal overexpression of *dpp *does not induce apoptosis at stage 14. (H) Excessive cell death was detected in embryos overexpressing dpp using *24B-gal4 *at stage 16. Click here for file

Additional file 4 **Fig. S4**. The expression of the endogenous *tkv *was specifically silenced as compared to *rps17 *mRNA in the embryos. RT-PCR products were resolved in 1.5% agarose gel and visualized with EtBr. WT, wild-type embryos. Click here for file

Additional file 5 **Fig. S5**. Ectodermally, but not mesodermlly overexpression of *raw *suppresses the ectopic pMad in *raw *mutant. (A) pMad was detected as a broad dorsal band in *raw *mutant (brackets). (B) Targeted expression of *raw *using *69B-gal4 *inhibited ectopic pMad in *raw *mutant. (C) Forced expression of *raw *using *24B-gal4 *can not inhibit ectopic pMad in *raw *mutant (brackets). Click here for file
