# Supplementary material for: Excessive Dpp signaling induces cardial apoptosis through dTAK1 and dJNK during late embryogenesis of Drosophila
Source: J Biomed Sci. 2011 Nov 24;18(1):85. doi: 10.1186/1423-0127-18-85 (PMC3247863; doi:10.1186/1423-0127-18-85)
Supplement: Additional file 1 — Fig. S1. Odd-skipped pericardial cells (OPCs) are missing in raw1 mutant embryos at late stages. (A) wild-type embryos show the presence of OPCs. (B) OPCs are completely absent in raw mutants. [file 1423-0127-18-85-S1.PDF]

Additional File 1

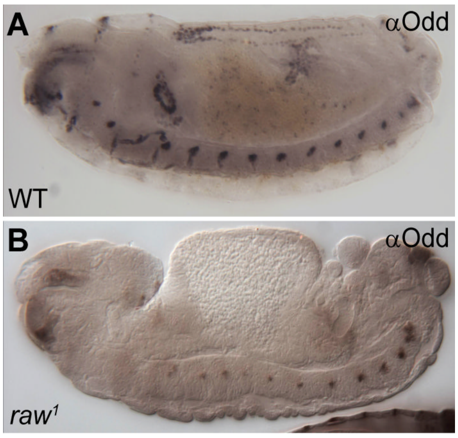

Fig. S1. Odd-skipped pericardial cells (OPCs) are missing in *raw*^{1} mutant embryos at late stages. (A) wild-type embryos show the presence of OPCs. (B) OPCs are completely absent in *raw* mutants.
