# Supplementary material for: Excessive Dpp signaling induces cardial apoptosis through dTAK1 and dJNK during late embryogenesis of Drosophila
Source: J Biomed Sci. 2011 Nov 24;18(1):85. doi: 10.1186/1423-0127-18-85 (PMC3247863; doi:10.1186/1423-0127-18-85)
Supplement: Additional file 2 — Fig. S2. Loss-of Wg function does not lead to localized apoptosis. (A, D) Wg was expressed in a series of ectodermal cells at dorsal and ventral sites of embryos at stage 13. The expression pattern was not altered in raw mutant. (B, E). Lateral expression of Wg became a transverse stripe in the dorsal ectoderm of wild-type embryos. However, its expression decreased significantly in raw mutant embryos at stage 14. (C, F) At stage 16, residual Wg staining was detected in the dorsal epidermis of wild-type embryos, but its expression was completely lost in raw mutants at stage 16. (G) raw mutation shows cadial apoptosis phenotype (brackets). (H) wgIL114 is a temperature-sensitive allele that mimics the null wg allele at non-permissive temperatures. Removal of wg function does not lead to cardial apoptosis phenotype in temperature shift experiment using wgIL114 allele. Incubation times (9-15 hr) were normalized to development at 25°C. (I) Ectopic wg expression driven by 69B-gal4, did not suppress cardial apoptosis in raw mutants (brackets). [file 1423-0127-18-85-S2.PDF]

Additional File 2

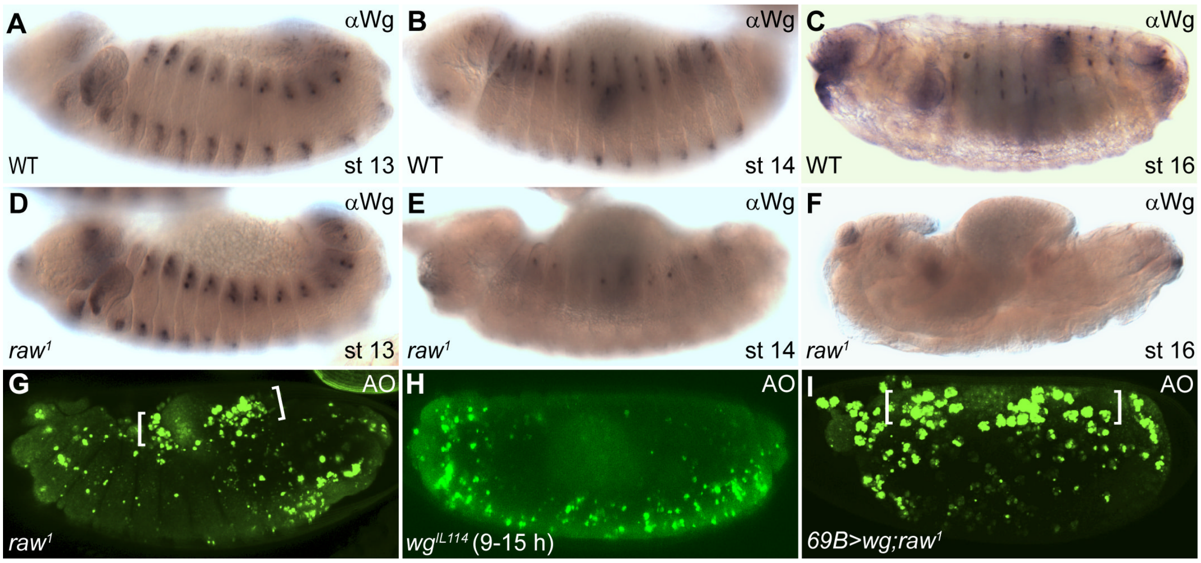

Fig. S2. Loss-of Wg function does not lead to localized apoptosis. (A, D) Wg was expressed in a series of ectodermal cells at dorsal and ventral sites of embryos at stage 13. The expression pattern was not altered in *raw* mutant. (B, E). Lateral expression of Wg became a transverse stripe in the dorsal ectoderm of wild-type embryos. However, its expression decreased significantly in *raw* mutant embryos at stage 14. (C, F) At stage 16, residual Wg staining was detected in the dorsal epidermis of wild-type embryos, but its expression was completely lost in *raw* mutants at stage 16. (G) *raw* mutation shows cadial apoptosis phenotype (brackets). (H) wg^{IL114} is a temperature-sensitive allele that mimics the null *wg* allele at non-permissive temperatures. Removal of *wg* function does not lead to cardial apoptosis phenotype in temperature shift experiment using wg^{IL114} allele. Incubation times (9-15 hr) were normalized to development at 25°C. (I) Ectopic *wg* expression driven by *69B-gal4*, did not suppress cardial apoptosis in *raw* mutants (brackets).
