# Supplementary material for: Excessive Dpp signaling induces cardial apoptosis through dTAK1 and dJNK during late embryogenesis of Drosophila
Source: J Biomed Sci. 2011 Nov 24;18(1):85. doi: 10.1186/1423-0127-18-85 (PMC3247863; doi:10.1186/1423-0127-18-85)
Supplement: Additional file 3 — Fig. S3. Mesodermally overexpression of Dpp induces raw-like phenotypes. (A,) him-GFP reporter was expressed in muscle and heart precursors in 24B-gal4 control flies at stage 14. (B) Expression of him-GFP was limited in heart cells in control 24B-gal4 driver at stage 16. (C) Mesodermal overexpression of dpp induced ectopic heart cells at stage 14. (D) him-GFP expressing heart cells were lost in embryos expressing dpp using 24B-gal4 at stage 16. (E, F) Normal AO staining pattern was observed in 24B-gal4 control driver at stage 14 and 16. (G) Mesodermal overexpression of dpp does not induce apoptosis at stage 14. (H) Excessive cell death was detected in embryos overexpressing dpp using 24B-gal4 at stage 16. [file 1423-0127-18-85-S3.PDF]

Additional File 3

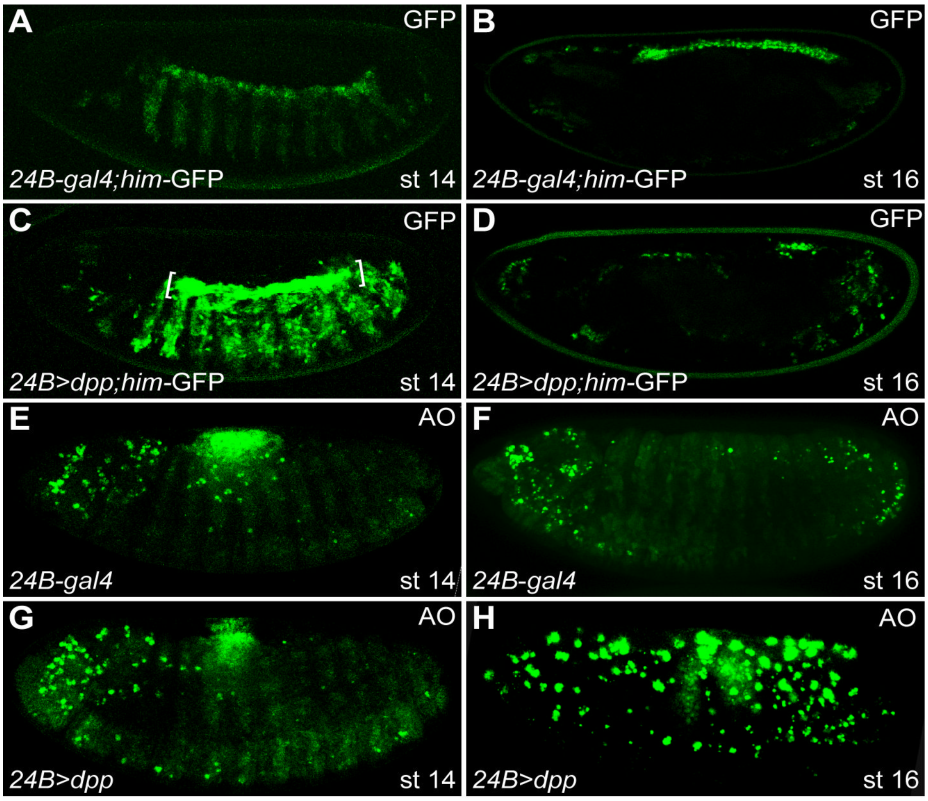

Fig. S3. Mesodermally overexpression of Dpp induces *raw*-like phenotypes. (A,) *him-GFP* reporter was expressed in muscle and heart precursors in *24B-gal4* control flies at stage 14. (B) Expression of *him-GFP* was limited in heart cells in control 24B-*gal4* driver at stage 16. (C) Mesodermal overexpression of *dpp* induced ectopic heart cells at stage 14. (D) *him-GFP* expressing heart cells were lost in embryos expressing *dpp* using *24B-gal4* at stage 16. (E, F) Normal AO staining pattern was observed in *24B-gal4* control driver at stage 14 and 16. (G) Mesodermal overexpression of *dpp* does not induce apoptosis at stage 14. (H) Excessive cell death was detected in embryos overexpressing dpp using *24B-gal4* at stage 16.
