# Supplementary material for: Excessive Dpp signaling induces cardial apoptosis through dTAK1 and dJNK during late embryogenesis of Drosophila
Source: J Biomed Sci. 2011 Nov 24;18(1):85. doi: 10.1186/1423-0127-18-85 (PMC3247863; doi:10.1186/1423-0127-18-85)
Supplement: Additional file 4 — Fig. S4. The expression of the endogenous tkv was specifically silenced as compared to rps17 mRNA in the embryos. RT-PCR products were resolved in 1.5% agarose gel and visualized with EtBr. WT, wild-type embryos. [file 1423-0127-18-85-S4.PDF]

Additional File 4

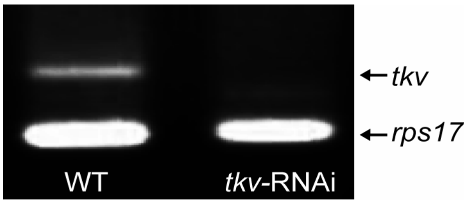

Fig. S4. The expression of the endogenous *tkv* was specifically silenced as compared to *rps17* mRNA in the embryos. RT-PCR products were resolved in 1.5 % agarose gel and visualized with EtBr. WT, wild-type embryos.
