# Supplementary material for: Excessive Dpp signaling induces cardial apoptosis through dTAK1 and dJNK during late embryogenesis of Drosophila
Source: J Biomed Sci. 2011 Nov 24;18(1):85. doi: 10.1186/1423-0127-18-85 (PMC3247863; doi:10.1186/1423-0127-18-85)
Supplement: Additional file 5 — Fig. S5. Ectodermally, but not mesodermlly overexpression of raw suppresses the ectopic pMad in raw mutant. (A) pMad was detected as a broad dorsal band in raw mutant (brackets). (B) Targeted expression of raw using 69B-gal4 inhibited ectopic pMad in raw mutant. (C) Forced expression of raw using 24B-gal4 can not inhibit ectopic pMad in raw mutant (brackets). [file 1423-0127-18-85-S5.PDF]

Additional File 5

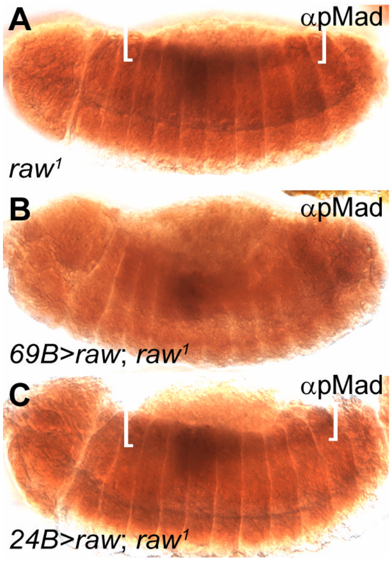

Fig. S5. Ectodermally, but not mesodermlly overexpression of *raw* suppresses the ectopic pMad in *raw* mutant. (A) pMad was detected as a broad dorsal band in *raw* mutant (brackets). (B) Targeted expression of *raw* using *69B-gal4* inhibited ectopic pMad in *raw* mutant. (C) Forced expression of *raw* using *24B-gal4* can not inhibit ectopic pMad in *raw* mutant (brackets).
